# Beyond cardiovascular disease: remnant cholesterol as a novel risk factor for osteoarthritis

**DOI:** 10.3389/fnut.2025.1692833

**Published:** 2025-11-17

**Authors:** Rui Xie, Zeping Chen, Guimin Zhang, Wei Zhao

**Affiliations:** 1 Department of Tuina, Chengdu Pidu District Hospital of Traditional Chinese Medicine, Chengdu, China; 2 Chengdu University of Traditional Chinese Medicine, Chengdu, China

**Keywords:** remnant cholesterol, osteoarthritis, ELSA, cohort study, BMI – body mass index

## Abstract

**Background:**

One of the main causes of impairment in older people globally is osteoarthritis (OA). The importance of metabolic variables in the pathophysiology of OA has received more attention than only mechanical stress. Triglyceride-rich lipoprotein remnants’ cholesterol component, remnant cholesterol (RC), has been linked to a number of metabolic and inflammatory diseases. Its relationship to the risk of OA is yet unknown, though. With an emphasis on the mediating function of body mass index (BMI), the research prospectively investigated the connection of RC levels with incident OA in middle-aged as well as older persons, drawing on data from the English Longitudinal Study of Ageing (ELSA).

**Methods:**

Participants free of OA at baseline were included. RC levels were estimated via the formula: triglycerides/2.2 (mmol/L). The outcome was newly diagnosed OA during follow-up. Cox proportional hazards models were used to examine the association between RC levels and incident OA. The models were adjusted for a range of potential confounders, including age, sex, race, education level, marital status, income, smoking status, alcohol frequency, physical activity level, and chronic comorbidities. Restricted cubic splines (RCS) were leveraged to evaluate dose–response connection. Subgroup analyses tested the robustness of the findings, and bootstrap-based mediation analysis evaluated the indirect effect of BMI.

**Results:**

Among 2,205 participants, 1,100 incident OA cases were identified during a median follow-up of 13.6 years. Higher RC levels were independently related with higher OA risk (highest vs. lowest quartile: HR = 1.27, 95% CI: 1.07–1.52; per unit increment: HR = 1.01, 95% CI: 1.01–1.03). RCS analysis showed a linear dose–response connection (*P* for nonlinearity >0.05). Subgroup analyses yielded consistent results without significant interactions (all *P*-interaction >0.05). Mediation analysis indicated BMI substantially mediated the RC–OA association, accounting for 84% of the effect.

**Conclusion:**

In this large prospective cohort of middle-aged and older adults, RC showed a positive, dose–response association with incident osteoarthritis that attenuated to near-null after adjustment for BMI. Mediation analysis indicated that approximately 84% of the total association operated via BMI, supporting adiposity as the principal pathway and suggesting limited BMI-independent effect of RC. These findings highlight RC as a potentially modifiable metabolic biomarker and underscore the interplay of dyslipidemia and obesity in OA pathogenesis, suggesting that RC management combined with weight control may offer an effective strategy for OA prevention.

## 1 Introduction

OA is a major cause of disability among middle-aged as well as older adults, with prevalence steadily rising alongside global population aging. Currently, OA affects nearly 595 million people, accounting for 7.6% of the global population, and is expected to increase further, imposing heavy healthcare and socioeconomic burdens (1–3). Existing treatments, including nonsteroidal anti-inflammatory drugs, rehabilitation, and joint replacement, mainly target symptom relief or late-stage structural damage, underscoring the need for early preventive strategies (4).

Although traditionally viewed as a degenerative “wear-and-tear” disorder, emerging evidence indicates that OA involves complex interactions between mechanical stress, metabolic abnormalities, lipid dysregulation, and low-grade systemic inflammation (5). Disrupted lipid metabolism may accelerate OA both indirectly, through systemic inflammation (6–9), and directly, via lipid accumulation and extracellular matrix disruption (10–12).

Remnant cholesterol (RC) —the cholesterol content of triglyceride-rich lipoprotein remnants such as VLDL, chylomicron remnants, and IDL—can be estimated from lipid profiles (e.g., triglycerides/2.2, mmol/L) (13, 14). Elevated RC has been strongly linked to atherosclerotic cardiovascular disease (15–17), renal dysfunction (18), and possibly chronic inflammatory joint diseases such as rheumatoid arthritis (19). However, its role in OA remains unexplored, and no large-scale prospective evidence is available. Given the shared mechanisms of lipid dysregulation and chronic inflammation in OA, investigating RC may reveal novel insights into OA pathogenesis and provide early metabolic targets for risk stratification and prevention.

Body mass index (BMI), reflecting both adiposity and mechanical load, is an established risk factor for OA (20). Beyond joint overloading, obesity promotes chronic inflammation, lipid disturbances, and abnormal adipokine secretion (5). Importantly, RC levels are strongly associated with obesity phenotypes (21), suggesting BMI may mediate the link between RC and OA. Quantifying this mediation is essential to disentangle direct metabolic effects from obesity-driven mechanisms and to guide integrated prevention strategies.

To address these gaps, we leveraged data from the *English Longitudinal Study of Ageing* (ELSA), a nationally representative prospective cohort that continuously tracks the health, social, and metabolic profiles of community-dwelling adults aged 50 years and older in England (22). This well-characterized dataset provides detailed biochemical and lifestyle measures, making it ideal for exploring the metabolic determinants of musculoskeletal diseases. Therefore, using data from the ELSA, this study prospectively examines the association between RC and incident OA, evaluates potential dose–response patterns, and quantifies the mediating role of BMI. These analyses aim to provide novel evidence on the metabolic contributions to OA development and inform early prevention strategies in ageing populations.

## 2 Methods

### 2.1 Study design and population

**Figure 1:**
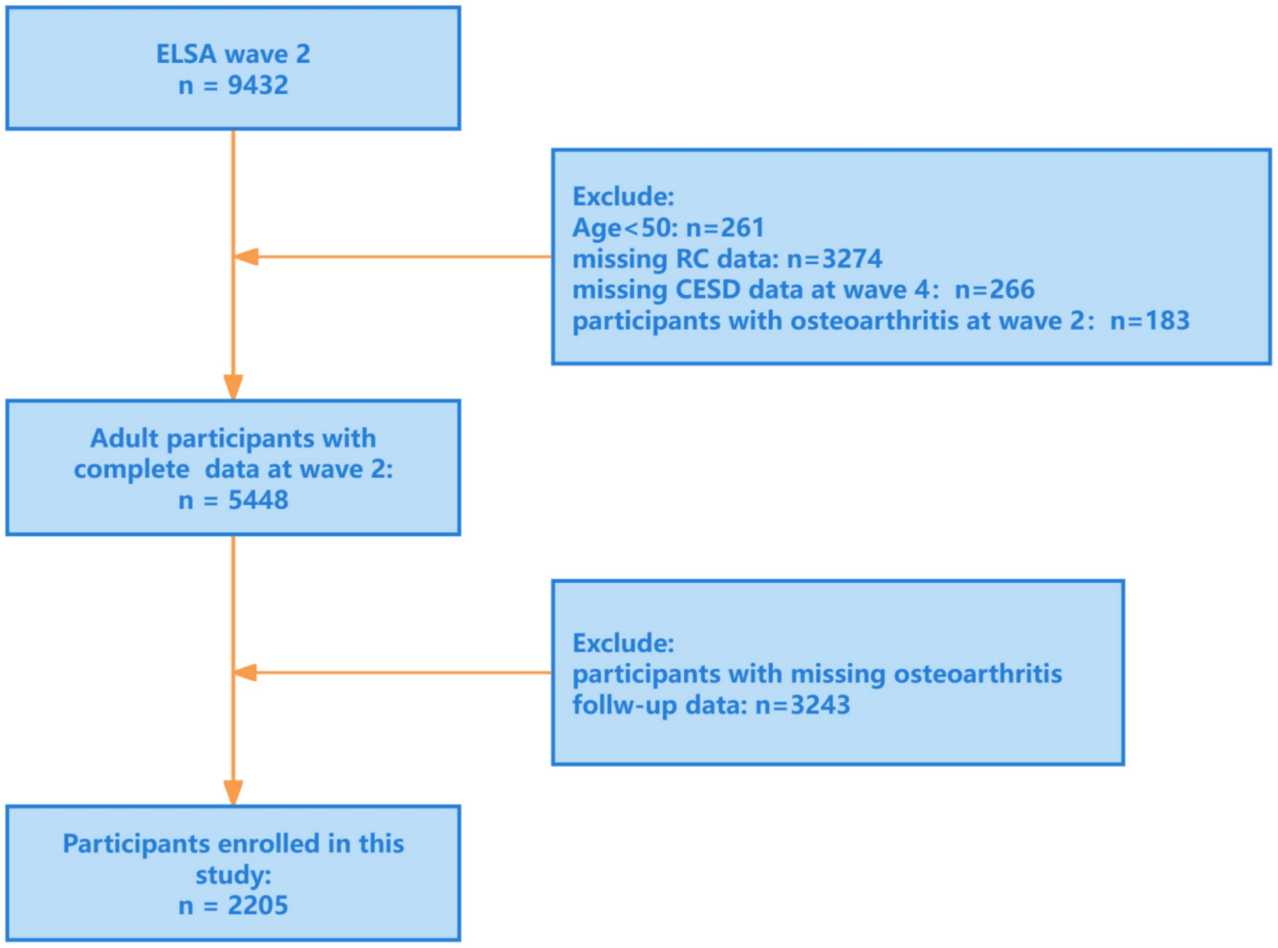
Research flowchart. ELSA, English Longitudinal Study of Ageing; RC, remnant cholesterol; CESD, the Center for Epidemiologic Studies Depression Scale (CES-D).

The research was a prospective cohort analysis based on the ELSA, a nationally representative cohort that has followed community-dwelling adults aged ≥50 years since 2002, collecting detailed information on health, socioeconomic status, and lifestyle (22). We defined Wave 2 (2004–2005) as baseline and included participants who were free of osteoarthritis (OA) at baseline, with follow-up through Wave 9 (2018–2019). After applying inclusion as well as exclusion criteria, 2,205 participants aged ≥50 years were included in the final analysis (Figure 1).

### 2.2 Variable definitions and measurements

#### 2.2.1 Remnant cholesterol

In accordance with current guidelines, since LDL-C was estimated, RC was calculated as triglycerides/2.2 (mmol/L) (13, 14).

#### 2.2.2 BMI

BMI was computed as weight in kilograms divided by height in meters squared (kg/m^2^).

#### 2.2.3 OA

Incident OA was identified at each ELSA wave using self-reported physician diagnosis from standardized questionnaires. Validation evidence indicates that self-reported OA has acceptable diagnostic accuracy at the population level (pooled sensitivity = 0.75 and specificity = 0.89), supporting its use when clinical examination is infeasible in large cohort studies (23).

### 2.3 Potential confounders

We considered a range of potential confounders which may affect the connection of RC with OA, including:

Demographic factors: age, sex, ethnicity, education, marital status, household income.

Lifestyle factors: smoking status (yes/no), alcohol consumption (≥1 time/week vs. <1 time/week), physical activity level (high/low).

Health status: chronic comorbidities such as hypertension, diabetes, and hyperlipidemia.

Missing data were addressed via multiple imputation by chained equations with random forest algorithms.

### 2.4 Statistical analysis

Categorical variables were reported as counts as well as percentages, whereas continuous variables were summarized as mean ± SD or median with interquartile range. *t*-tests or Kruskal-Wallis tests for continuous variables as well as χ^2^ tests for categorical data were leveraged to assess for differences between groups. Plotting of Kaplan–Meier survival curves was done, and the log-rank test was leveraged to evaluate any differences.

Cox proportional hazards regression models were applied to examine the association between RC and incident OA, with results presented as hazard ratios (HRs) and 95% confidence intervals (CIs). To enhance interpretability, a stepwise modeling strategy was adopted:

Model 1: Unadjusted model, showing the crude association between RC and OA.

Model 2: Adjusted for demographic covariates, including age, gender, race, marital, education, income.

Model 3: Further adjusted for clinical and lifestyle variables, including hypertension, diabetes, cancer, CHD, depression, hyperlipidemia, smoking, alcohol use, and physical activity status.

Stratified subgroup analyses by age, sex, education, marital status, smoking, alcohol use, BMI, and physical activity were performed to evaluate robustness.

Mediation analysis was carried out via the “mediation” package in R to assess the indirect effect of BMI on the RC–OA association. All confounders were adjusted, and the proportion mediated was estimated using the bootstrap method with 1,000 replications to obtain 95% CIs. Statistical tests were two-sided, with a significance level of *α* = 0.05.

## 3 Results

### 3.1 Baseline characteristics

**Figure 2:**
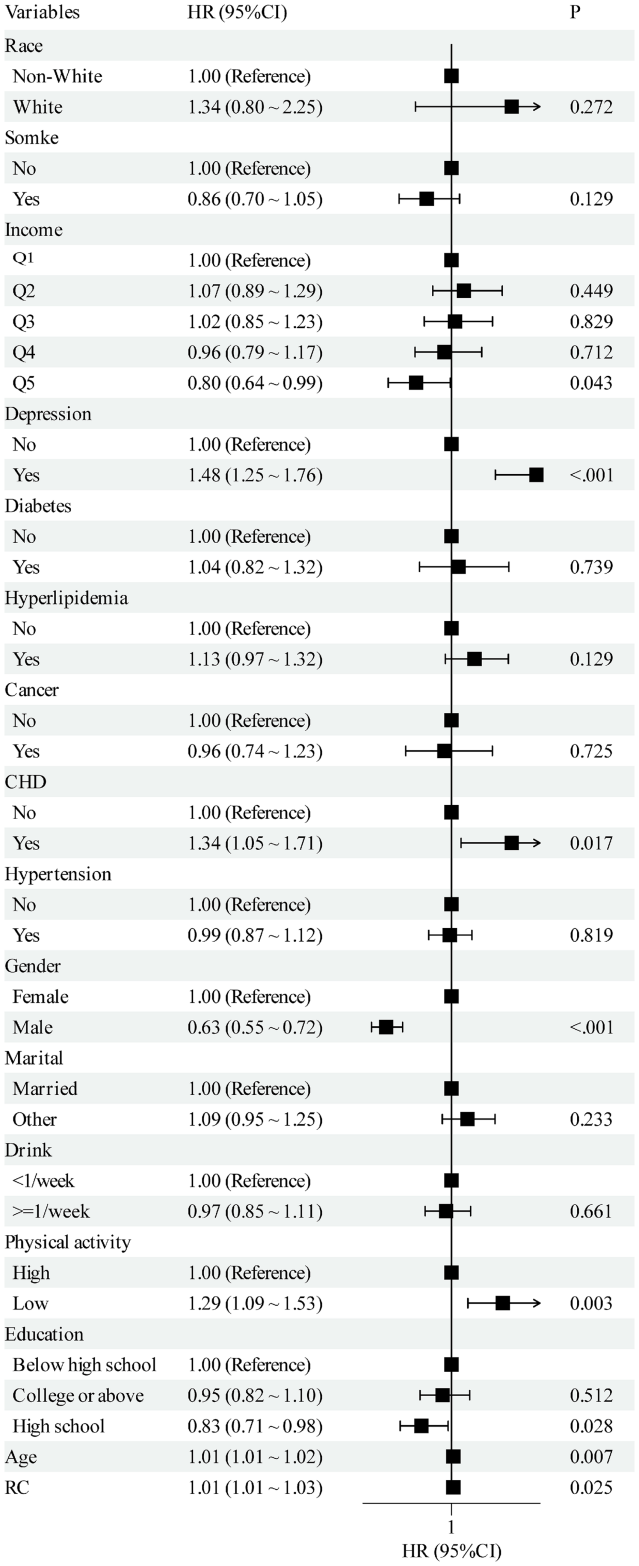
Multivariate forest plot of the association between RC and OA. RC, remnant cholesterol; OA, osteoarthritis; CHD, coronary heart disease.

2,205 participants were incorporated, with a mean age of 62 years; 43.8% were men, 98% were White, and 88.4% were married. The mean baseline RC level was 2.71 mmol/L. During a median follow-up of 163 months, 1,100 incident OA cases were identified (Table 1). Additionally, among the variables included as baseline characteristics and covariates in the adjusted models, depression, coronary heart disease (CHD), sex, physical activity, high-school education level, and age were each significantly and positively associated with incident OA. Detailed estimates are provided in the Figure 2.

**Table 1:** Baseline characteristics of study populations across OA.

Variable	Levels	*N*	Overall	Absence of OA	Presence of OA	*p*-value
			*N* = 2,205	*N* = 1,105	*N* = 1,100	
Age, mean (SD)		2,205	62.16 (6.98)	61.47 (6.89)	62.86 (7.01)	<0.001
BMI, mean (SD)		2,205	27.80 (4.75)	27.06 (4.23)	28.55 (5.12)	<0.001
RC (mmol/L), mean (SD)		2,205	2.71 (4.57)	2.38 (3.72)	3.05 (5.27)	<0.001
Follow-up time (month), (SD)		2,205	115.79 (62.06)	167.53 (3.19)	63.80 (48.13)	<0.001
Race, *n* (p%)		2,205				0.320
	Non-White		36.00 (1.63%)	21.00 (1.90%)	15.00 (1.36%)	
	White		2,169.00 (98.37%)	1,084.00 (98.10%)	1,085.00 (98.64%)	
Gender, *n* (p%)		2,205				<0.001
	Female		1,240.00 (56.24%)	518.00 (46.88%)	722.00 (65.64%)	
	Male		965.00 (43.76%)	587.00 (53.12%)	378.00 (34.36%)	
Marital, *n* (p%)		2,205				0.001
	Married		1,584.00 (71.84%)	828.00 (74.93%)	756.00 (68.73%)	
	Other		621.00 (28.16%)	277.00 (25.07%)	344.00 (31.27%)	
Education, *n* (p%)		2,205				<0.001
	Below high school		703.00 (31.88%)	304.00 (27.51%)	399.00 (36.27%)	
	College or above		976.00 (44.26%)	528.00 (47.78%)	448.00 (40.73%)	
	High school		526.00 (23.85%)	273.00 (24.71%)	253.00 (23.00%)	
Smoke, *n* (p%)		2,205				0.193
	No		1,953.00 (88.57%)	969.00 (87.69%)	984.00 (89.45%)	
	Yes		252.00 (11.43%)	136.00 (12.31%)	116.00 (10.55%)	
Drink, *n* (p%)		2,205				<0.001
	<1/week		713.00 (32.34%)	318.00 (28.78%)	395.00 (35.91%)	
	≥1 week		1,492.00 (67.66%)	787.00 (71.22%)	705.00 (64.09%)	
Physical activity, *n* (p%)		2,205				<0.001
	High		1,925.00 (87.30%)	1,000.00 (90.50%)	925.00 (84.09%)	
	Low		280.00 (12.70%)	105.00 (9.50%)	175.00 (15.91%)	
Income (5th quintile), *n* (p%)		2,205				<0.001
	Q1		441.00 (20.00%)	205.00 (18.55%)	236.00 (21.45%)	
	Q2		441.00 (20.00%)	181.00 (16.38%)	260.00 (23.64%)	
	Q3		441.00 (20.00%)	209.00 (18.91%)	232.00 (21.09%)	
	Q4		441.00 (20.00%)	232.00 (21.00%)	209.00 (19.00%)	
	Q5		441.00 (20.00%)	278.00 (25.16%)	163.00 (14.82%)	
Diabetes, *n* (p%)		2,205				0.252
	No		2,060.00 (93.42%)	1,039.00 (94.03%)	1,021.00 (92.82%)	
	Yes		145.00 (6.58%)	66.00 (5.97%)	79.00 (7.18%)	
Hyperlipidemia, *n* (p%)		2,205				0.078
	No		1,837.00 (83.31%)	936.00 (84.71%)	901.00 (81.91%)	
	Yes		368.00 (16.69%)	169.00 (15.29%)	199.00 (18.09%)	
Cancer, *n* (p%)		2,205				0.208
	No		2,088.00 (94.69%)	1,053.00 (95.29%)	1,035.00 (94.09%)	
	Yes		117.00 (5.31%)	52.00 (4.71%)	65.00 (5.91%)	
Coronary heart disease, *n* (p%)		2,205				0.027
	No		2,077.00 (94.20%)	1,053.00 (95.29%)	1,024.00 (93.09%)	
	Yes		128.00 (5.80%)	52.00 (4.71%)	76.00 (6.91%)	
Hypertension, *n* (p%)		2,205				0.340
	No		1,165.00 (52.83%)	595.00 (53.85%)	570.00 (51.82%)	
	Yes		1,040.00 (47.17%)	510.00 (46.15%)	530.00 (48.18%)	
Depression, *n* (p%)		2,205				<0.001
	No		1,967.00 (89.21%)	1,026.00 (92.85%)	941.00 (85.55%)	
	Yes		238.00 (10.79%)	79.00 (7.15%)	159.00 (14.45%)	

OA, osteoarthritis.

### 3.2 Association between RC and OA

**Figure 3:**
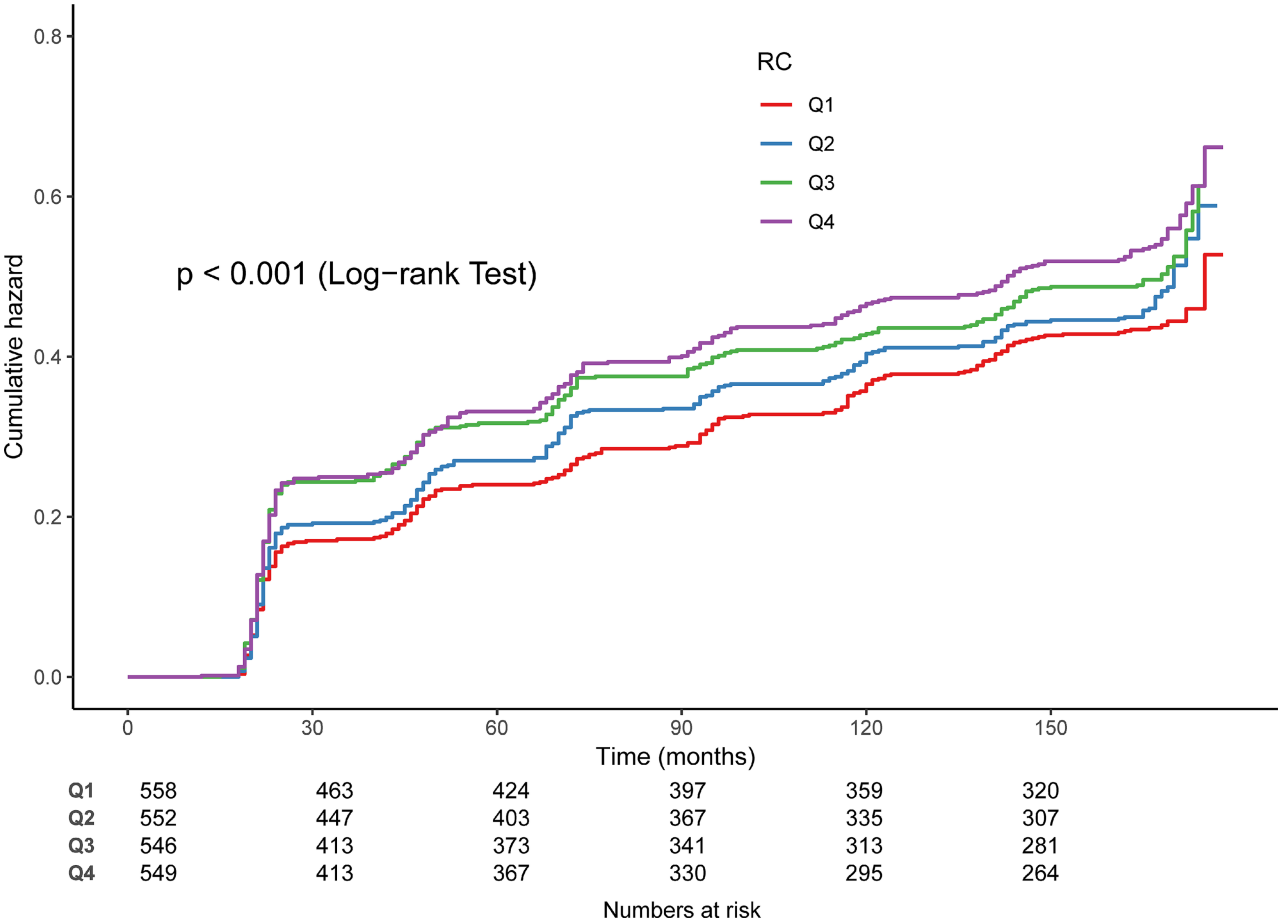
Kaplan–Meier curves for the cumulative incidence of OA.

**Figure 4:**
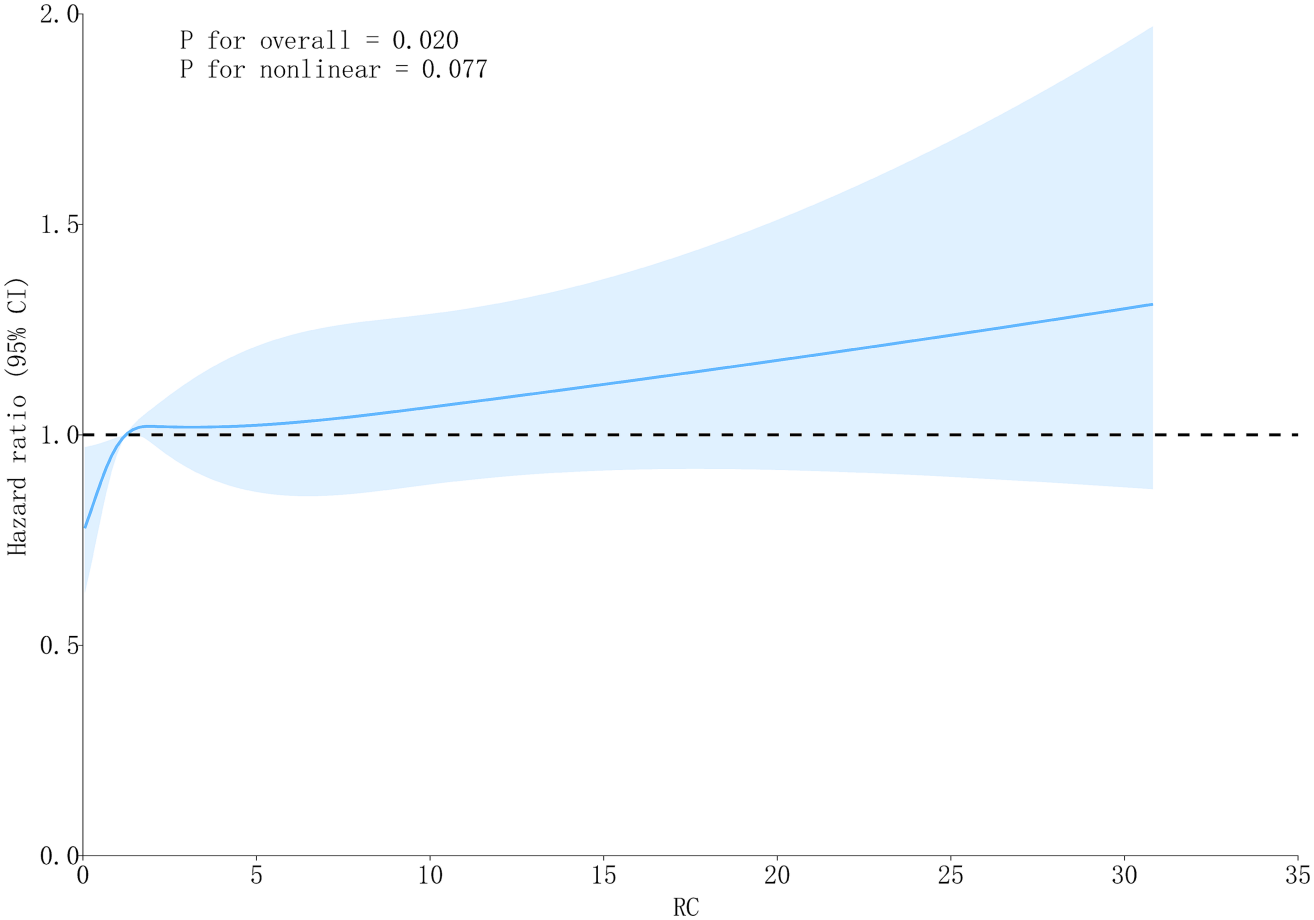
The RCS curve of the association between RC and OA.

In Cox regression models, RC as a continuous variable was positively related to OA risk. Each 1 mmol/L rise in RC corresponded to approximately a 1–2% higher risk of OA (fully adjusted model: HR = 1.01, 95% CI: 1.01–1.03) (Table 2). When RC was categorized into quartiles, results were consistent: compared with the lowest quartile (Q1), participants in Q3 and Q4 had significantly higher risk (HR = 1.22, 95% CI: 1.02–1.45; HR = 1.27, 95% CI: 1.07–1.52, respectively). Kaplan–Meier curves demonstrated higher cumulative incidence of OA in participants with elevated RC (Figure 3). RCS analysis suggested a linear dose–response connection of RC with OA, with no evidence of nonlinearity (*P*-nonlinearity = 0.108) (Figure 4).

**Table 2:** Correlation between RC and the risk of OA.

Variables	Model 1		Model 2		Model 3	
	HR (95%CI)	*P*	HR (95%CI)	*P*	HR (95%CI)	*P*
RC	1.02 (1.01 ~ 1.03)	**<0.001**	1.02 (1.01 ~ 1.03)	**0.003**	1.01 (1.01 ~ 1.03)	**0.028**
RC quartile						
Q1	1.00 (Reference)		1.00 (Reference)		1.00 (Reference)	
Q2	1.18 (0.99 ~ 1.40)	0.068	1.18 (0.99 ~ 1.40)	0.069	1.17 (0.99 ~ 1.40)	0.072
Q3	1.25 (1.05 ~ 1.49)	**0.011**	1.22 (1.03 ~ 1.45)	**0.025**	1.22 (1.02 ~ 1.45)	**0.030**
Q4	1.42 (1.19 ~ 1.68)	**<0.001**	1.32 (1.11 ~ 1.57)	**0.001**	1.27 (1.07 ~ 1.52)	**0.008**
*P* for trend		**<0.001**		**0.006**		**0.036**

HR, hazard ratio; CI, confidence interval.

Model 1: Crude.

Model 2: Adjust: Age, Gender, Race, Marital, Education, Income.

Model 3: Adjust: Age, Gender, Race, Education, Income, Marital, Physical activity, Smoke, Drink, Diabetes, Hyperlipidemia, Cancer, CHD, Hypertension, Depression.The bolded values indicate that the *P*-values are less than 0.05.

**Figure 5:**
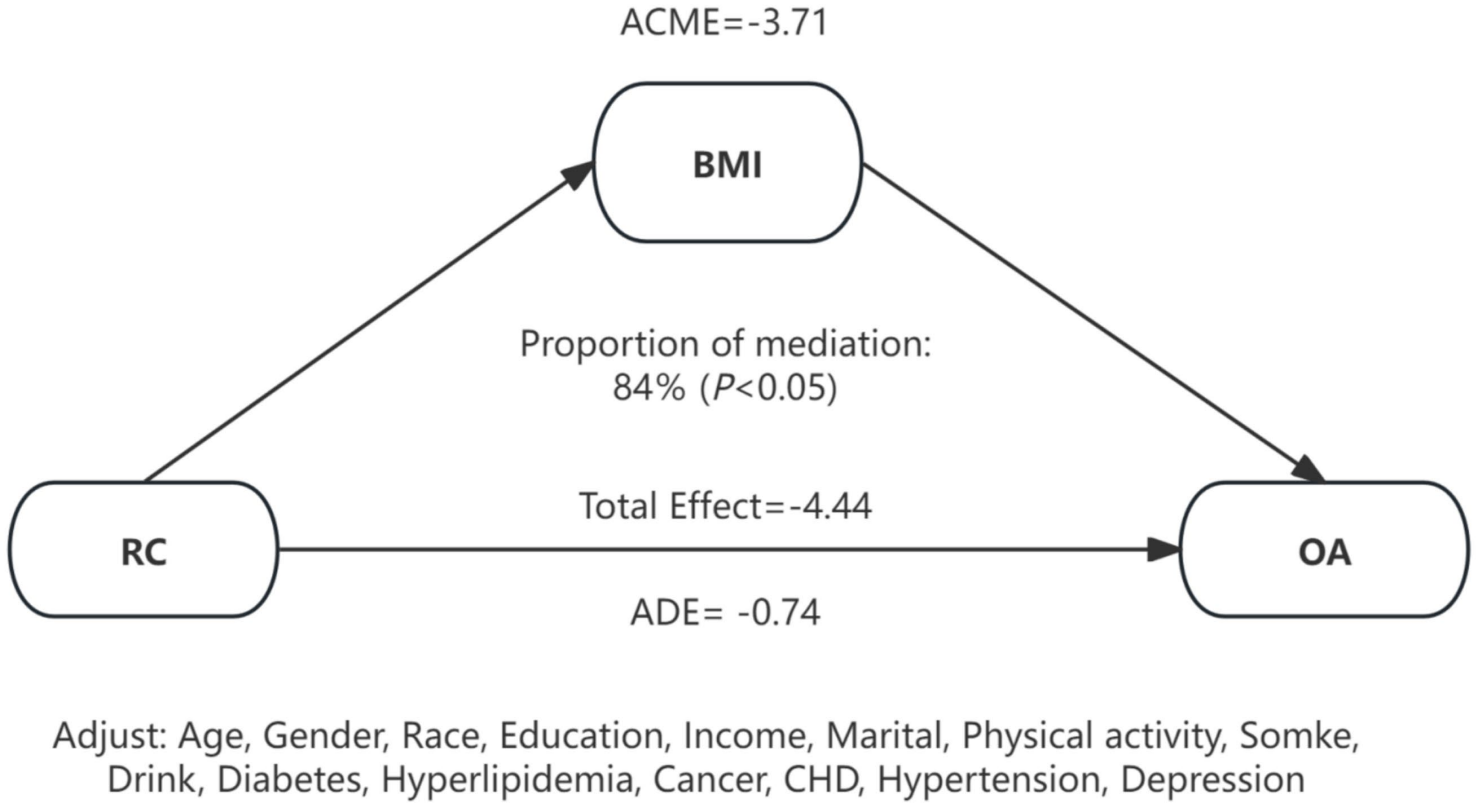
Mediation effects of BMI on the association between RC and OA. RC, remnant cholesterol; OA, osteoarthritis; BMI, body mass index; CHD, coronary heart disease; ACME, average causal mediation effect; ADE, average direct effect.

Because BMI is hypothesized to lie on the causal pathway linking RC to OA, our primary Cox models did not include BMI to estimate the total effect of RC. To further examine whether an independent association persisted after accounting for BMI, we additionally fit Models (Model 3 + BMI) to estimate the direct effect. After BMI adjustment, the association between RC and OA was substantially attenuated and became non-significant (HR = 1.00, 95% CI: 0.99–1.02). This attenuation pattern is consistent with BMI acting as a major mediator through adiposity-related mechanisms such as mechanical loading and low-grade systemic inflammation. In mediation analysis, BMI statistically accounted for approximately 84% of the total association between RC and OA (*p* < 0.05) (Figure 5), supporting the hypothesis that RC influences OA risk primarily through BMI-dependent pathways. In sex-stratified analyses, the mediating role of BMI differed by sex. Among males, BMI statistically mediated approximately 62% of the association between RC and OA (*p* < 0.05), whereas no significant mediation effect was observed in females (*p* > 0.05).

### 3.3 Subgroup analyses

**Figure 6:**
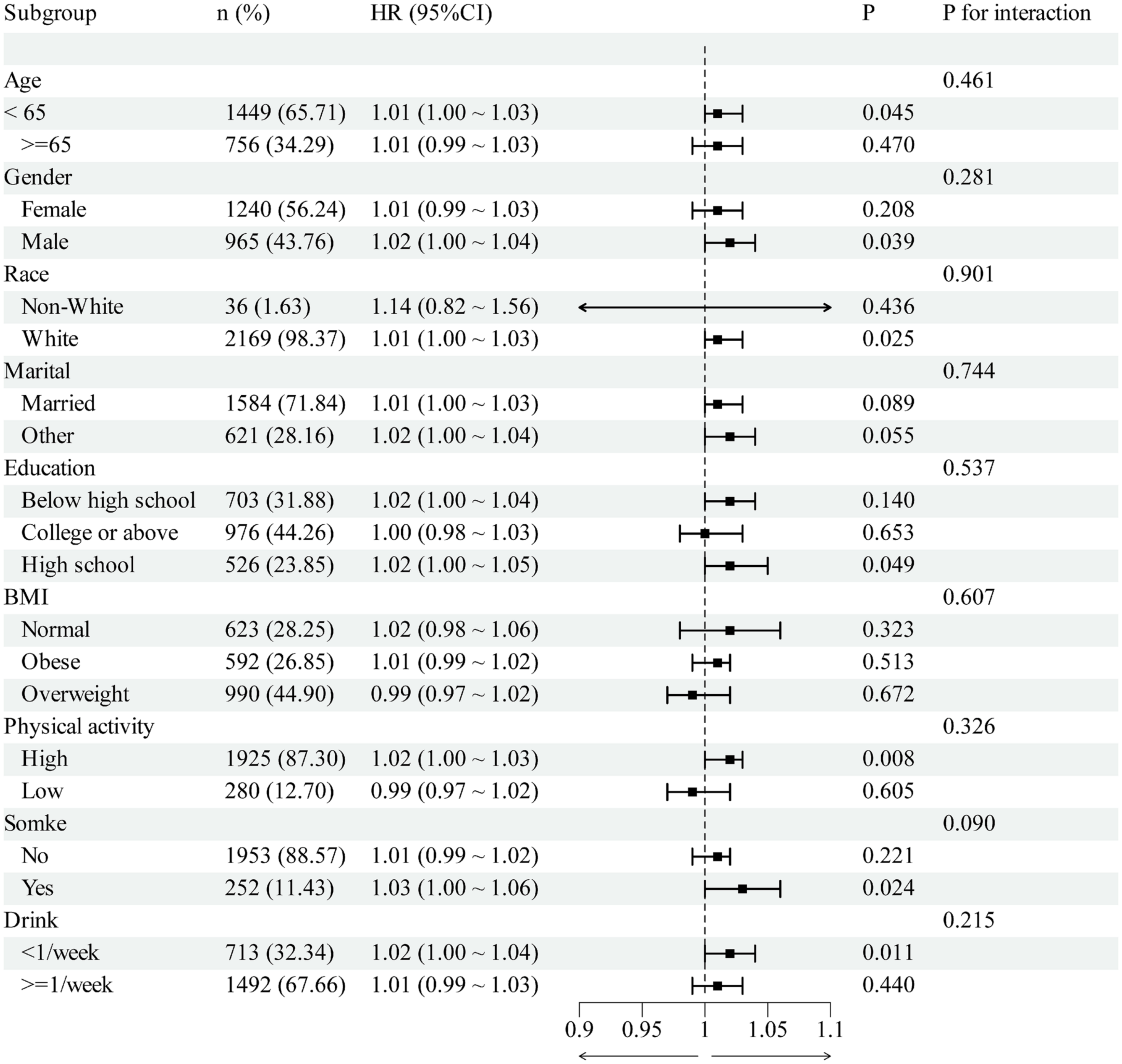
The association between RC and OA in different subgroups.

Subgroup analyses were nominally significant in some strata (age <65, male, White, high-school education, high physical activity, <1 drink/week); however, interaction tests were non-significant for all subgroups (all *P* interaction >0.05). Notably, in the obese subgroup, no clear association was observed for RC per 1-unit increase (Figure 6).

## 4 Discussion

Based on the large prospective ELSA cohort, the research is the first to systematically assess the connection between RC and incident OA in middle-aged as well as older adults. We found higher RC was related with a significantly higher risk of OA in a linear dose–response manner. Furthermore, mediation analysis indicated that BMI accounted for a substantial proportion of this association, suggesting that both metabolic and mechanical pathways may jointly contribute to OA development. However, these mediation estimates should be interpreted with caution: our observational design is susceptible to residual confounding, and validity requires no unmeasured confounding of the exposure–mediator–outcome pathways and no mediator–outcome confounders affected by exposure. While we tested and, if needed, modeled exposure–mediator interaction, the results remain explanatory rather than strictly causal.

Without adjustment for BMI, RC was positively associated with incident OA (HR 1.01, 95% CI 1.01–1.03). After adding BMI to the Cox model, the association attenuated to non-significance (HR 1.00, 95% CI 0.99–1.02). This pattern is compatible with adiposity-related pathways—mechanical loading and low-grade inflammation—suggesting that BMI may lie on the RC → OA pathway. To separate pathway mediation from metric attenuation due to the non-collapsibility of Cox hazard ratios, we prespecified and reported the total effect (without BMI) alongside the direct effect (with BMI), and conducted counterfactual mediation. The mediation analysis indicated that approximately 84% of the total RC–OA effect operated via BMI, with only a small BMI-independent component. Consistent with this interpretation, tests for interaction across BMI categories (normal, overweight, obese) were non-significant, and in the obese subgroup we observed no clear association between RC and incident OA; these subgroup findings are exploratory given multiplicity and unequal precision. Taken together, these findings support adiposity as the principal conduit linking RC to OA and imply limited independent predictive value of RC beyond BMI. We note that mediation proportions in non-linear models are scale-dependent and that single baseline measurements of RC and BMI may bias estimates toward the null.

Obesity and elevated BMI have long been recognized as major risk factors for OA, acting not only through mechanical loading but also via chronic low-grade inflammation and lipid disturbances (24). Consistent with this, growing evidence has highlighted the role of lipid dysregulation in OA. Epidemiological studies have reported associations between dyslipidemia and OA, although the direction and strength of associations for specific lipid fractions (e.g., HDL-C, LDL-C) vary across populations and study designs (25–27). For example, a UK Biobank study reported a paradoxical causal relationship whereby genetically lower LDL-C predisposed individuals to higher OA risk (28, 29), challenging conventional assumptions and suggesting instability of causal evidence (30). Clinical observations further support the link between metabolic imbalance and OA phenotypes, as obesity as well as hyperlipidemia are related with more severe synovitis, structural damage, and worse functional outcomes (31). Adipokines such as leptin have also been implicated in cartilage damage and disease progression (32, 33), reinforcing the “metabolism–inflammation–cartilage degradation” pathway.

Prospective evidence on “nontraditional lipid markers and OA” remains limited, and large-scale data on RC in particular have been lacking. Previous work has focused mainly on rheumatoid arthritis, showing higher RC is related with higher RA incidence and cardiovascular risk in RA patients (19, 34, 35). Our study extends this evidence to OA, demonstrating for the first time in a large aging cohort that elevated RC independently predicts incident OA, thus broadening RC from a cardiovascular and inflammatory arthritis biomarker to a metabolic risk indicator relevant for degenerative joint disease. Notably, our mediation analysis further quantified the chain “RC → BMI → OA,” providing evidence that metabolic dysfunction may act synergistically with obesity to drive OA risk (36).

RC—the cholesterol cargo of triglyceride-rich lipoprotein (TRL) remnants (37)—provides a biologically coherent link to OA through lipid–metabolic and inflammatory pathways. TRL remnants can traverse the endothelium and become retained in the subendothelial space; macrophage ApoE-mediated uptake of VLDL/remnants induces an M1 pro-inflammatory phenotype, foam-cell formation, and up-regulation of inflammatory mediators (accompanied by activation of matrix metalloproteinases, etc.) (38–40). These processes are presumed to extend to the joint microvasculature and synovium (41, 42).

In inflamed milieus, redox-modified lipoproteins such as oxidized LDL directly accelerate cartilage matrix loss and inflammatory chondrocyte death by disrupting TFEB-regulated autophagy–lysosome function and engaging NF-κB programs (5, 43, 44). OA cartilage also exhibits lipid-metabolic reprogramming, with enhanced fatty-acid oxidation shown to drive disease progression in experimental models (12, 45). Complementary lipidomic studies report phospho- and sphingolipid enrichment in synovial fluid/tissues, aligning systemic dyslipidaemia with local joint lipid remodeling (46, 47).

Finally, consistent with recent metabolic and osteoarthritis literature, RC correlates positively with adiposity and insulin resistance (21, 48, 49), while obesity promotes OA through adipokine-mediated low-grade inflammation and mechanical loading (33, 50, 51); together, these observations support a biologically plausible “RC → (BMI/metabolic dysregulation) → OA” axis and align with the substantial BMI-explained proportion observed in our study.

Moreover, higher physical activity related to lower OA risk, consistent with its anti-inflammatory effects, weight control, and improved joint homeostasis (52, 53). Depressive symptoms were associated with higher OA risk, potentially via HPA-axis activation, systemic inflammation, pain amplification, and reduced activity (54, 55). CHD showed a positive association, underscoring shared cardio-metabolic-inflammatory pathways with OA and the burden of multimorbidity (56, 57). These covariates were included primarily to control confounding; their estimates are descriptive and reported transparently.

Under standard mediation assumptions, BMI statistically explained ~84% of the RC–OA association. Mechanistically, RC co-varies with adiposity and insulin resistance, while adiposity contributes to OA via mechanical loading and adipo-inflammatory signaling. Taken together, these data support a pathway in which RC predominantly relates to OA through BMI-linked mechanisms, with a small residual BMI-independent component. We interpret these estimates as explanatory rather than strictly causal, acknowledging residual confounding and single-time measurements.

In clinical terms, the BMI-independent predictive value of RC for OA appears modest, consistent with evidence that RC tracks adiposity and insulin resistance and associates with adverse obesity phenotypes in population studies (21, 48). Nevertheless, RC may add value to multivariable risk profiling, particularly in metabolically unhealthy phenotypes or normal-BMI individuals with dyslipidaemia, where BMI alone incompletely captures metabolic inflammation and ectopic fat. RC also denotes modifiable risk: unhealthy lifestyle patterns (smoking, low physical activity, poor diet) associate with higher RC (58), and lipid-lowering trials show that reductions in remnant cholesterol accompany lower cardiovascular risk (59, 60), supporting RC as a tractable target within the metabolic–inflammatory axis that is biologically relevant to OA. To define RC’s independent utility for OA, prospective studies with repeated measurements, interventions aimed at remnant lowering, and causal designs (e.g., Mendelian randomization) are warranted (61, 62).

There are several limitations warrant mention. First, we acknowledge that single baseline assessments of RC/BMI and wave-based OA ascertainment limit our ability to implement a precise 2–3-year time-lag. Consequently, reverse causation cannot be fully excluded. Given potential regression-dilution from one-time exposure measurement and the absence of exact event dates, our estimates should be viewed as conservative associations. The finding that BMI statistically explains a large proportion of the RC–OA association under mediation assumptions is consistent with scenarios in which subclinical disease at baseline and adiposity-related pathways co-evolve. Future datasets with exact diagnosis dates and repeated biomarker assessments will allow formal landmark/delayed-entry analyses to more directly address reverse causation. Second, OA diagnosis relied primarily on self-report or physician report without systematic imaging verification, which may introduce recall bias—exacerbated by symptom fluctuations—and non-differential misclassification (63, 64). Third, lack of imaging-based phenotyping limited our ability to explore structural subtypes and progression. Future studies should incorporate longitudinal imaging and inflammatory biomarkers to clarify mechanisms and evaluate the impact of RC-lowering interventions on OA risk.

## 5 Conclusion

In a large UK cohort of middle-aged and older adults, the RC–osteoarthritis association became non-significant after BMI adjustment and appeared largely BMI-mediated. Our results highlight the intertwined role of metabolic–mechanical pathways, with BMI serving as a key mediator. These results suggest that RC may represent a new, modifiable metabolic biomarker for OA and provide new evidence supporting the “metabolism–obesity–OA” pathway. Early RC management, together with weight control, could serve as dual intervention targets for OA prevention.

## Data Availability

The original contributions presented in the study are included in the article/supplementary material, further inquiries can be directed to the corresponding author.
